# Construction of stably maintained non-mobilizable derivatives of RSF1010 lacking all known elements essential for mobilization

**DOI:** 10.1186/1472-6750-7-80

**Published:** 2007-11-21

**Authors:** Joanna I Katashkina, Tatiana M Kuvaeva, Irina G Andreeva, Alexandra Yu Skorokhodova, Irina V Biryukova, Irina L Tokmakova, Lubov I Golubeva, Sergey V Mashko

**Affiliations:** 1 Closed Joint-Stock Company "Ajinomoto-Genetika Research Institute", 1^st ^Dorozhny Pr. 1, Moscow 117545, Russia; 2 GosNIIgenetika, State Federal Unitary Enterprise, 1^st ^Dorozhny Pr. 1, Moscow 117545, Russia

## Abstract

**Background:**

RSF1010 is a well-studied broad-host-range plasmid able to be mobilized to different bacteria and plants. RSF1010-derived plasmid vectors are widely used in both basic research and industrial applications. In the latter case, exploiting of mobilizable plasmids or even the plasmids possessing negligible mobilization frequency, but containing DNA fragments that could promote conjugal transfer, is undesirable because of biosafety considerations. Previously, several mutations significantly decreasing efficiency of RSF1010 mobilization have been selected. Nevertheless, construction of the RSF1010 derivative lacking all known loci involved in the conjugal transfer has not been reported yet.

**Results:**

Novel non-mobilizable derivatives of RSF1010 lacking all known DNA sequences involved in the mobilization process have been obtained due to the exploiting of λRed-driven recombination between the plasmid and a constructed *in vitro *linear DNA fragment. To provide auto-regulated transcription of the essential replication gene, *repB*, the plasmid loci *oriT*, *mobC *and *mobA *were substituted by the DNA fragment containing P_*lac*UV5_→*lacI*. Mobilization of the obtained RSFmob plasmid was not detected in standard tests. The derivative of RSFmob with increased copy number has been obtained after *lacI *elimination. High stability of both constructed plasmids has been demonstrated in *Escherichia coli *and *Pantoea ananatis*. Design of RSFmob allows easy substitution of P_*lac*UV5 _by any desirable promoter for construction of novel derivatives with changed copy number or host range.

**Conclusion:**

Novel non-mobilizable derivatives of RSF1010 lacking all known DNA sequences involved in the mobilization process and stably maintained at least in *E. coli *and *P. ananatis *have been constructed. The obtained plasmids became the progenitors of new cloning vectors answering all biosafety requirements of genetically modified organisms used in scale-up production.

## Background

The first sequence of bacterial chromosome was published more than 10 years ago. Now, hundreds of bacterial genomes are available for comparative genomics. The vast genetic information as well as introduction of new bacterial strains to industrial usage require the suitable tools for rapid genetic manipulations with a wide variety of bacterial species. Broad-host-range plasmids provide a valuable tool for such work. Numerous broad-host-range shuttle vectors, promoter-probe vectors, expression vectors and other special-purpose vectors are available now. The majority of them are based on the replicons of the IncQ or IncP groups [see for example [1-3]].

Undoubtedly, RSF1010 can be considered as the most studied plasmid of this type. RSF1010 is a mobilizable, non-conjugative, IncQ group plasmid that has broad host range replication properties in most of the Gram^-^ bacteria [4] and at least some of the Gram^+ ^species [5]. RSF1010 can use many different transfer systems for its mobilization. This property enables it to be transferred to a wide variety of bacterial hosts and even to plants [6]. The nucleotide sequence of this plasmid has been determined [7] and the detailed functional study of all plasmid loci involved in replication and mobilization has been performed [see for review [4,8,9]]. Relatively small size (8684 bp), moderate copy-number (in *E. coli*, RSF1010 is present at a copy number of 12 per cell [10]), capability to replicate and stable maintenance in a broad range of bacterial hosts where RSF1010 could be easily transferred from the standard laboratory *E. coli *strains by conjugative mobilization, have made this plasmid an attractive molecular cloning vector for basic research.

On the other hand, RSF1010 was traditionally used in biotechnology. It is very suitable vector for gene amplification in bacterial producing strains because of its high stability in different hosts. Moreover, in *E. coli *it is compatible with other popular replicons such as pBR322, pSC101, etc. Due to this property, RSF1010 was used as a second and even third plasmid vector in the real industrial strains producing different metabolites. However, industrial usage of mobilizable plasmids is undesirable because of biosafety considerations and is restricted today by legislations of several leading countries described, for example, in "Guidelines for research involving recombinant DNA molecules" published by the NIH on the 7th of May, 1987 and in the European Council Directives 90/220/EEC, 98/81/EC and 90/219/EEC. That is why construction of the stably maintained mob^- ^derivative of RSF1010 (the derivative carrying mutation(s) in *mob *locus, which decreases mobilization frequency significantly) could have important practical application. The modern advanced methods of bacterial transformation, especially the electro transformation technique, provide a simple way for introduction of plasmid DNA to a broad range of bacterial hosts, which is not dependent on conjugation and mobilization [see e.g. [11,12] and for review [13,14]]. Thus, in principle, mob^- ^derivatives of RSF1010 could be used not only in *E. coli*, but also in other bacterial hosts supporting RSF1010 replication for that methods of chemical or electro transformation have been elaborated.

Previously, several mutations of RSF1010 have been selected and characterized that significantly decreased efficiency of the plasmid mobilization in the standard laboratory tests [15]. But these mutations inactivate only few from the total set of genetic loci of RSF1010 that participate in mobilization process, and so, from the formal point of view the corresponding plasmids could not be considered as non-mobilizable for non-restricted practical application in the industrial biotechnology. All known genetic loci involved in mobilization have to be eliminated from the officially safe RSF1010-derivatives.

**Figure 1:**
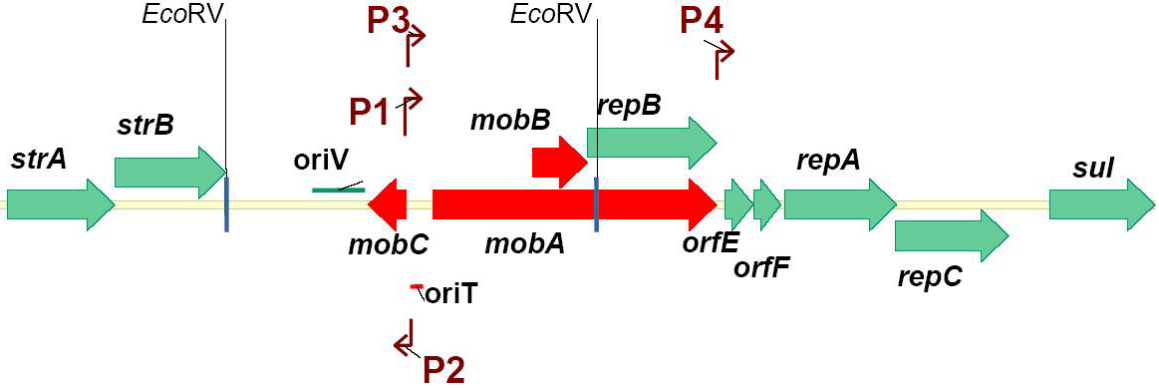
**Map of the plasmid RSF1010**. The loci involved in conjugal transfer are in red.

At present, the following loci involved in the plasmid vegetative replication and its mobilization, have been identified in RSF1010 [16,17] (see Fig. 1 as well):

1) Transcription of all corresponding genes is initiated from the promoters (P1 – P4) (see Fig. 1). It is under multiple regulatory controls, and the individual plasmid-encoded proteins are involved, as a rule, in several functions essential for plasmid replication, mobilization and regulation of transcription. P1 and P3 controlled by MobC and MobA provide transcription of *mobA/repB, mobB*, and, probably, (*E-F-repA-repC*)-operon. The P2-promoter is under the same auto-regulated control and provides transcription of *mobC *in the opposite direction. The P4 promoter providing transcription of (*E-F-repA-repC*)-operon, is under the auto-regulated control of the F-repressor, whose gene is located in the same operon [18]. Earlier it was shown [15] that deletion of the P4 promoter does not lead to the loss of the plasmid "availability": transcription of *repB (repB') *– (*E-F-repA-repC*)-operon from the P1/P3 promoters is sufficient for the plasmid replication;

*2) oriV *– the unique origin of vegetative DNA replication;

*3) repA, repB *(alternative designation that more precisely reflects the function of its protein product – *mobA/repB*), and *repC *– the genes for essential replication proteins. Hereat, *repA *and *repC *encode a helicase and an *oriV *binding protein, correspondingly, and the expression level of *repC*, in particular, regulates the extent of plasmid replication. The product of *mobA/repB *– RepB is a multifunctional protein where its C-terminal domain, RepB', exhibits DNA primase activity in the process of vegetative plasmid replication, whereas its N-terminal part, MobA, is essential for the plasmid mobilization, and as well as MobC, plays a role in transcription regulation of the plasmid genes;

*4) oriT *– the site of relaxation complex and origin of conjugative DNA transfer;

Activities of MobA, MobB and MobC providing the specific cleavage of plasmid DNA in *oriT *followed by mobilizable transfer, are essential for mobilization of RSF1010. MobC encoded by *mobC*, in cooperation with MobA are co-regulators of of plasmid gene transcription, participating, in particular, in auto-regulation of their own synthesis. The *mobB *gene encoding MobB, is located in the structural part of *mobA/repB*, but translation of the corresponding proteins is occurred in the different reading frames.

The auto-regulated expression of RSF1010 genes referred to above provides the control of plasmid copy number, its stable maintenance, vegetative replication and mobilization. The corresponding genetic elements are partially overlapped in a rather complicated fashion. All recently obtained RSF1010-based plasmids with partially deleted *mob*-genes possessed mobilization frequencies detectable even under laboratory conditions. Besides, most of the mutant plasmids had significantly increased copy number [15].

For construction of RSF1010 derivatives that could be officially considered as mob^-^, we have decided to substitute the plasmid fragment comprising *mobC*, *oriT *(overlapping with P1, P2 and P3) and 5'-terminal portion of *mobA/repB *encoding MobB and MobA, by an artificial DNA fragment that could provide efficient transcription and translation of *repB*. The earlier described [19] genetic element, P_***lac*UV5**_→*lacI *linked to sub-optimal SD sequence GGGGGG has been used initially for reproducing the auto-regulated expression pattern of the native *mobA/repB *gene.

## Results and discussion

### Construction of the RSFmob plasmid

As unique endonuclease restriction sites were absent in the target loci of RSF1010, we decided to apply a previously developed technique for plasmid gene replacement provided by Red-recombination system of phage λ [20]. Such an approach is extensively exploited for obtaining bacterial artificial chromosome rearrangements [21,22], but it is not routinely used for *in vivo *construction of multicopy recombinant plasmids.

**Figure 2:**
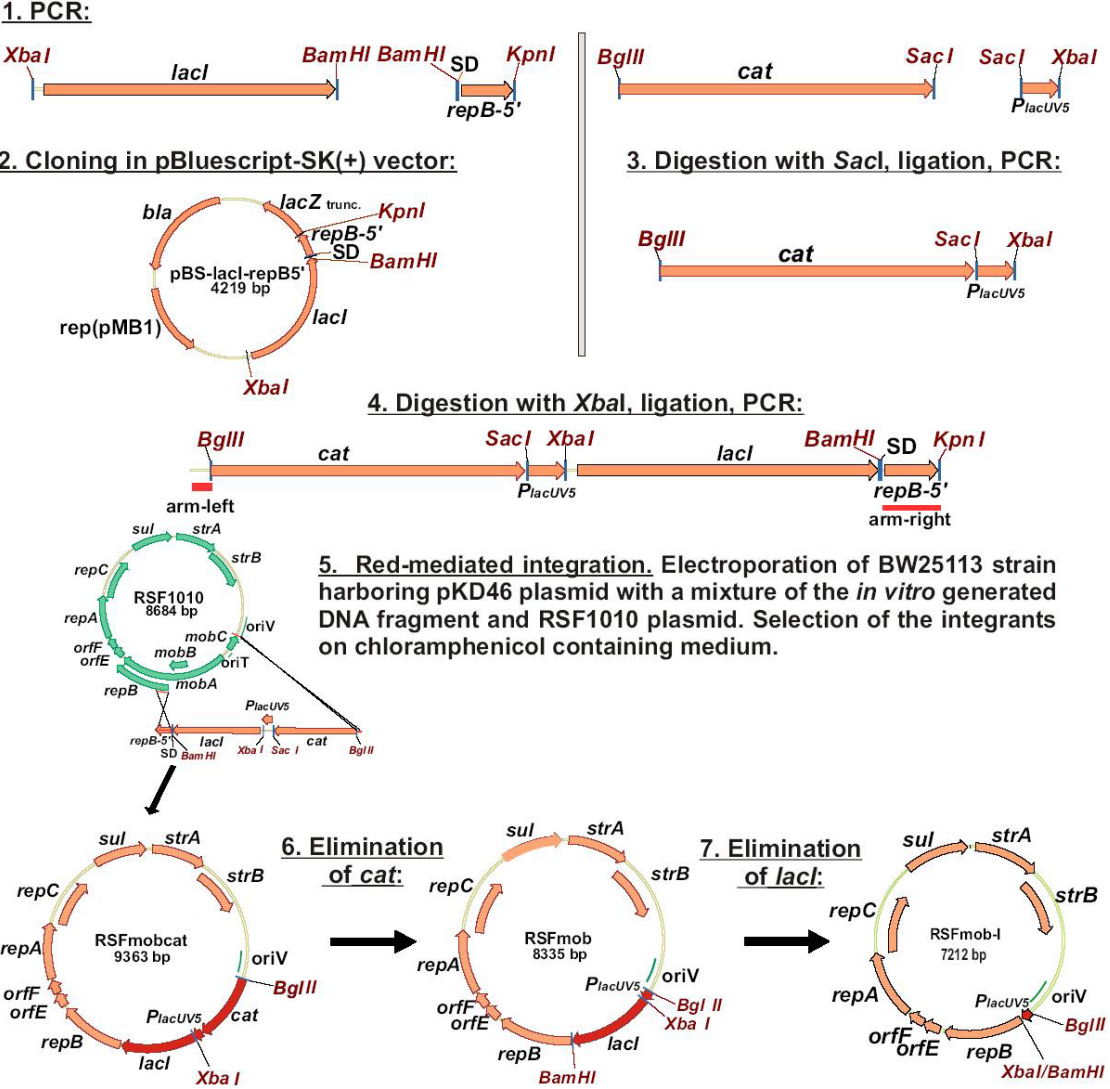
Construction of mob^- ^derivatives of RSF1010.

λRed-mediated substitution of RSF1010 *mob *locus by an *in vitro *generated DNA fragment containing an auto-regulated element P_***lac*UV5**_→*lacI *marked by chloramphenicol resistance gene (*cat*) and the flanks strictly homologous to the target sites on the plasmid, was performed (Fig. 2) and described in detail in Methods. *E. coli *K12 MG1655 bearing pKD46 helper plasmid [23] was used as a recipient strain for λRed-mediated integration of the constructed fragment to RSF1010.

Initially, we transformed the MG1655/pKD46 strain with RSF1010. The resultant bi-plasmid strain grown in arabinose-containing medium for induction of λRed genes was electroporated with the constructed DNA fragment. Plasmids were isolated from the Cm^R^-clones and re-transformed into *E. coli *TG1 strain. According to restriction analysis, all plasmids from re-transformants had unexpected structure. Their patterns showed the presence of the integrated *cat*-P_***lac*UV5**_→*lacI *fragment, but the rest part of the plasmid contained a DNA fragment corresponding to RSF1010 plasmid of the wild-type and some additional DNA fragments probably flanking the integrative cassette. Formation of the recombinant plasmids joined in tandem to nonrecombinant plasmids in the analogous experiment with pBR322 derivatives was described previously [20]. In *E. coli *cells, RSF1010 replicates by a strand displacement mechanism [24] and has about 12 copies [10]. As only one of them participates in recombination with the transformed DNA fragment, we propose that the selected plasmids could be the products of recombination (maybe, induced by λRed system) between the desirable integrant and the wild-type copies of RSF1010.

To avoid the possible undesirable secondary recombination events, we have modified the procedure by introducing the integrative DNA fragment to the cells simultaneously with RSF1010. To that, MG1655/pKD46 strain was electroporated with the mixture of the DNA fragment to be integrated and the RSF1010 plasmid DNA (about 100 ng of each). Plasmid DNA isolated from the Cm^R ^clone grown after the electroporation, was re-transformed to *E. coli *TG1. The obtained Cm^R ^transformant contained the expected plasmid named RSFmobcat (GenBank accession number EF467358).

To remove the *cat *gene, the isolated RSFmobcat plasmid was digested with BglII and XbaI followed by ligation of the largest fragment (without *cat *and P_***lac*UV5**_) with BglII-XbaI fragment of the P_***lac*UV5 **_promoter. The later fragment was generated after hydrolysis by the mentioned endonucleases of the DNA amplified in polymerase chain reaction with the primers P1 and P8 and the plasmid pMW-P_lacUV5_-lacI-118 [19] as a template. The resulting plasmid was designated RSFmob (GenBank accession number EF467359).

### Mobilization frequency of the RSFmob plasmid

The mobilization efficiency of RSFmob as compared to the parental RSF1010 was tested. For this purpose, donor strains were constructed on the basis of *E. coli *C600 (r^+^m^+^leu^-^) transformed with the RP1.2 (Tc^R^) [25]. This plasmid carried the *tra*-operon necessary for conjugal transfer. RSF1010 and RSFmob were transformed into C600 [RP1.2] using *str*AB as a selective marker. The constructed strains were used as the donors in conjugation experiments with the recipient strain MG1655(leu^+^) to determine the mobilization efficiency. The experiment showed that RSFmob had an undetectable level of mobilization frequency (less than 10^-7^) that was at the minimum 5 orders of magnitude lower than for mobilizable RSF1010 (10^-2^). The described earlier[15] Δ13, Δ18 and Δ20 mutations in the *mob *locus of RSF1010 decreased its mobilization frequency up to 4.0 × 10^-6^, 1.7 × 10^-6 ^and 9.0 × 10^-8^, respectively.

### Evaluation of plasmid copy number

**Figure 3:**
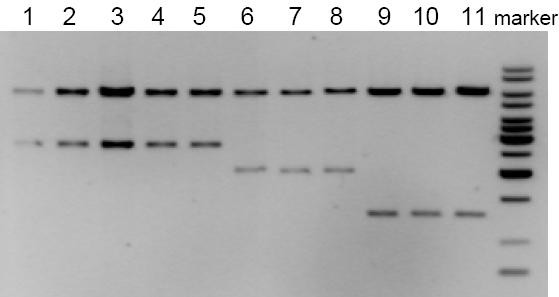
**Estimation of the relative copy numbers of the RSFmob and RSFmob-I plasmids**. RSF1010, RSFmob and RSFmob-I plasmids were transformed to *E. coli *MG1655 strain. Three independently obtained transformants of each type were analyzed. 1, 2, 3 – 0.5, 2.0 and 5.0 μl of the DNA probe isolated from clone 1 of MG1655/RSF1010; 4, 5 – 2.0 μl of the DNA probes isolated from clones 2 and 3 of MG1655/RSF1010; 6, 7, 8 – 2.0 μl of the DNA probes isolated from 3 independent clones of MG1655/RSFmob; 9, 10, 11 – 2.0 μl of the DNA probes isolated from 3 independent clones of MG1655/RSFmob-I. Relative copy number was calculated as the ratio of the DNA amounts in the upper bands (5.9 kb EcoRV-EcoRV fragment). Electrophoretic patterns for stationary phase are represented as an example.

Rough evaluation by electrophoresis of purified DNA (see, Methods) showed that RSFmob had approximately the same copy-number as RSF1010 in the stationary growth phase (Fig. 3, Table 1). During logarithmic growth, the copy-number of the obtained derivative was about two times lower than that of RSF1010. These data were in direct correlation with the levels of streptomycin resistance of the corresponding plasmid strains. Addition of 1 mM IPTG to the medium caused a 2.5-fold increase of RSFmob copy-number at the logarithmic stage of cell growing (Table 1). It was proposed that elimination of the *lacI *gene from the RSFmob could increase the plasmid copy-number due to derepression of *repB *and/or *repAC *transcription [26,27].

**Table 1:** Relative copy numbers of the constructed mob^- ^derivatives of the RSF1010 plasmid

**Plasmid**	**Relative copy number**			**Sm resistance, mg/L**
		
	**CT = 16 h**	**CT = 3 h**		
			
		**0 mM IPTG**	**1 mM IPTG**	
RSF1010	1.0 ± 0.3	1.0 ± 0.1	1.0 ± 0.1	450
RSF1010mob	0.9 ± 0.1	0.6 ± 0.1	1.4 ± 0.1	250
RSF1010mob-I	2.6 ± 0.3	1.8 ± 0.2	2.0 ± 0.1	700

Copy number of RSF1010 plasmid was taken as 1.0. Streptomycin resistance of the plasmid strains was determined on LB agar plates containing different concentrations of the antibiotic in 16 hours incubation at +37°C.

The derivative of RSFmob lacking the *lacI *gene was constructed due to the cleavage of the plasmid DNA isolated from MG1655dam^- ^(see Methods) by XbaI and BamHI followed by blunt-ending and circularization of the largest DNA fragment by T4 ligase. The constructed plasmid was designated RSFmob-I (GenBank accession number EF467360). Indeed, the copy-number of RSFmob-I was 3-fold higher than for RSFmob (see Fig. 3 and Table 1). Interestingly, the same increase in plasmid copy number had been observed previously for RSF1010-derivatives with inactivated *mobA *or *mobC *encoding the negative regulators of P1/P3 and P2 promoters [15].

### Stability of RSFmob-like plasmids in *Escherichia coli *and *Pantoea ananatis*

For testing the stability of the new plasmids in *E. coli*, seven 12-hour passages in LB medium of the *E. coli *MG1655 strain carrying RSFmob, RSFmob-I or RSF1010 were performed. Each culture grown under the non-selective conditions was plated on LB-agar followed by replica plating of 100 clones of each strain on the same medium with or without addition of streptomycin. Str^S^-clones were not detected among the tested ones. Thus, the frequency of plasmid loss was less than 1% after seven passages (more than 50 generations) under non-selective conditions in LB medium for the both obtained plasmids RSFmob and RSFmob-I, as well as for RSF1010.

Moreover, we have tested replication of RSFmob in *Pantoea ananatis*. Biotechnological companies are intensively investigating now this bacterium belonging to *Enterobacteriaceae *as a candidate host for industrial production of different useful metabolites [see e.g. [28,29]]. Our previous experiments showed that RSF1010 is stably maintained in this organism (data not shown). We introduced RSF1010 and RSFmob to *P. ananatis *SC17 strain by electroporation method (see Methods). Electroporation frequency was the same (about 10^5 ^plasmid clones per 10^8 ^survived recipient cells) for the both plasmid probes isolated from *E. coli *TG1 strain. For the both plasmids, the frequency of plasmid loss was less than 1% after four 12-hour passages (more than 50 generations) under non-selective conditions in LB medium. Therefore, RSFmob can replicate not only in *E. coli*, but also in *P. ananatis *and, probably, in other species related to *E. coli*.

As for the unrelated species, we have checked replication ability of RSFmob-I in *Methylophilus methylotrophus*. This obligate methylotroph is an interesting object of biotechnology [30]. Electroporation of RSFmob-I to *M. methylotrophus *AS1 strain (the wild-type strain, ATCC 53528, NCIMB 10515) was performed as in [30]; RSF1010 was used as positive control in this experiment. Sm^R ^clones were selected in the both cases, but isolation of plasmid DNA from them performed according to [30] has revealed presence of the plasmid only in the clones obtained after transformation with RSF1010. Therefore, RSFmob-I is unable to autonomous replication in *M. methylotrophus*. It was previously shown that the RepB' protein can substitute MobA to provide RSF1010 replication *in vitro *and *in vivo *in *E. coli *[24,15]. Moreover, co-expression of the *repB*', *repA *and *repC *genes provided *in trans *replication of the mini-plasmid carrying oriV_**RSF1010 **_in *Pseudomonas aeruginosa *[31]. The constructed mob^- ^derivatives of RSF1010 carry the coding parts of all three genes necessary for replication. Therefore, the most probable reason of the observed loss of replication ability in *M. methylotrophus *is insufficient expression of *repB*' resulting from low strength of the P_***lac*UV5 **_promoter in this bacterium. To solve this problem, P_***lac*UV5 **_or entire P_***lac*UV5**_→*lacI *element can be easily substituted by any convenient regulatory region recognized in the concrete bacterial host using unique BglII, XbaI and BamHI recognition sites specially introduced in the obtained plasmids. Novel mob^- ^derivatives of RSF1010 differing in copy number or host range can be easily generated from RSFmob in this way.

## Conclusion

Although the genetic loci responsible for the vegetative plasmid replication and mobilization are partially overlapped in the native structure of the well-studied broad-host-range plasmid RSF1010, we could obtain the new derivatives lacking all sequences participating in mobilization. These new plasmids do not only possess significantly decreased efficiency of mobilization (< 10^-7^) in the standard laboratory tests, but could be considered as non-mobilizable according to the formal requirements of the plasmids that could be used in the applied biotechnology.

The modified procedure of Red-driven recombination was exploited to provide the rather complicated substitution of the constructed *in vitro *artificial DNA fragment for the locus of the moderate copy number plasmid to be deleted. Such kind of the so-called recombineering experiments is widely used now for construction/modification of bacterial native/artificial chromosomes, but is not so popular for manipulation with high or moderate copy number plasmids.

Initially, the artificial auto-regulated genetic element, P_*lac*UV5_→*lacI*, was used to provide transcription of the genes responsible for vegetative replication of the plasmid. This element substituted the P1/P3 native promoters of RSF1010 that transcribe, in particular, the gene of their own repressor MobA [15]. This regulation is one of the factors controlling the copy number of the native RSF1010 plasmid. Exploiting of the artificial regulatory element for the same auto-regulation leads to stable maintaining of the new plasmid (for at least 50 generations) and save the plasmid copy number at the level of approximately two times lower, in comparison with RSF1010, at the logarithmic stage of growth, and practically the same level, as for RSF1010, in the stationary phase. Moreover, the copy number could be increased for the new RSFmob plasmid due to addition of IPTG in the culture medium. This dependence of the plasmid copy number on the presence of IPTG in the culture medium could be useful for exploiting of the new plasmid as the vector in the experiments on optimization of the cloned gene expression. Meanwhile, elimination of the *lacI *gene from RSFmob has led to construction of the new plasmid, RSFmob-I, that is stably maintained in *E. coli*, as well, and its copy number is 3-fold increased, in comparison with RSFmob. RSFmob contains specially introduced unique endonuclease recognition sites, which allow easy substitution of P_*lac*UV5_→*lacI *element by any desirable promoter or regulatory element for the possible changing of plasmid copy number and/or host range according to the aims of future experiments.

As for real exploiting of RSFmob-based recombinant plasmids in industrial strains, the set of further modifications have been provided recently in our laboratory that have been devoted to introducing of *thyA *as the selective marker instead of genes of antibiotic resistance (*strAB, sul*) [32].

## Methods

### Bacterial strains, plasmids and media

The bacterial strains and plasmids used in this work are listed in Table 2. *Escherichia coli *K-12 MG1655 strain transformed with the pKD46 plasmid was used as a recipient strain for λRed-mediated recombination. *E. coli *K12 TG1 was used for cloning and re-transformation of the plasmid mixtures obtained after λRed-mediated recombination. *E. coli *K12 C600 strain was used in the conjugal experiment. The specially obtained in this work *E. coli *MG1655dam^- ^strain was used for isolation of RSFmob plasmid DNA with unmethylated XbaI site necessary for construction of RSFmob-I. To obtain this strain, dam13::Tn9(Cm^R^) mutation was transferred to MG1655 from the streptomycin resistant strain GM2163 by P1-transduction. *P. ananatis *SC17, a low phlegm-producing mutant derived from the AJ13355-wild-type strain (see [28]) was kindly provided by Ajinomoto Co. BW25113/pKD46 strain kindly provided by Prof. B.L. Wanner was used as a donor of pKD46 plasmid. pBluescript II SK(+) was used as a cloning vector. pMW-P_***lac*UV5**_-lacI-118 from the laboratory collection was used as a template for PCR amplification of the P_***lac*UV5**_-*lacI *element. pACYC184 was used as a donor of the *cat *gene. RP1.2 plasmid carrying *tra *operon was used in mobilization experiments.

**Table 2:** The bacterial strains and plasmids used in this work

**Strain/Plasmid**	**Genotype/accession number**	**Source**	Reference
Strains			

Escherichia coli K-12 MG1655	Wild-type	The All-Russian Collection of Industrial Microorganisms (VKPM)	[35]
E. coli K-12 TG1	Δ(*lac-pro*) *supE thi hsdD5 F'traD36 proAB lacI*^q ^*lacZΔM15*	VKPM collection	[36]
*E. coli *K12 C600	F^- ^e14^- ^(McrA^-^) *thi-1 leuB6 lacY1 fhuA21 glnV44 rfbD1*	VKPM collection	[37]
*E. coli *K12 MG1655dam^-^	dam13::Tn9(Cm)	This work	
*E. coli *K12 GM2163	F-*ara*14 *leu*B6 *tonA*31 *lacY*1 *tsx*78 *supE*44 *galK*2 *galT*22 *hisG*4 *rpsL*136 *xyl*5 *mlt*1 *thi*1 *dam*13::Tn9(Cm) *dsm*6 *hsdR*2 *mcrB*1 *mcrA*	VKPM collection	
*E. coli *K12 BW25113	*lacI*^q ^*rrnB*_T14 _Δ*lacZ*_WJ16 _*hsdR*514 Δ*araBAD*_AH33 _ΔrhaBAD_LD78_	Prof. B.L. Wanner (personal communications)	[23]
*P. ananatis *SC17	A low phlegm-producing mutant strain	Ajinomoto Co	[28]
Plasmids			
RSF1010	GenBank accession number NC_001740	Ajinomoto Co	[7]
pBluescript II SK(+)	GenBank accession number X52328	Stratagene	
pMW-P_*lac*UV5_-lacI-118		From the laboratory collection	[19]
PACYC184	GenBank accession number X06403	Fermentas	
RP1.2		Drs. E.G. Abalakina and J.V. Jomantas (personal communications)	[25]
pKD46	GenBank accession number AY048746	Prof. B.L. Wanner (personal communications)	[23]

LB and SOC media were prepared as described in [33].

### PCR

AccuTaq LA DNA polymerase (Sigma) was used in accordance with the manufacturer's instructions to generate the DNA fragments for cloning and integration.

### Oligonucleotides

P1 5'-cctttggtaccagatctgcgggcagtgagcgcaacgc-3'

P2 5'-aattgggatccgctcactgcccgctttccagtcggg-3'

P3 5'-cgcttggatccggggggtggcccgatgaagaacgacag-3'

P4 5'-ctcttggtaccgcctgatatacacgtcattgcc-3'

P5 5'-tagcgagatctctgatgtccggcggtgcttttg-3'

P6 5'-aaaaagagctcttacgccccgccctgccactc-3'

P7 5'-cctttgagctcgcgggcagtgagcgcaacgc-3'

P8 5'-ctgtttctagatcctgtgtgaaattgttatccgc-3'

P9 5'-gcagggcctgtctcggtcgatcattcagcccggctcatagatctctgatgtccggcggtgc-3'

### Recombinant DNA technique

DNA manipulations were performed according to standard methods [33]. Restriction endonucleases and Klenow fragment of DNA polymerase I *E. coli *were from Fermentas, T4 DNA ligase was from Promega.

### Generation of the DNA fragment for λRed-mediated recombination

At first, a DNA fragment comprising structural part of the *lacI *gene under control of the P_***lac*UV5 **_promoter was amplified by PCR using primers P1 and P2 and the pMW-P_***lac*UV5**_-lacI-118 plasmid [19] as a template. Primer P1 annealed to the 5'-terminus of the P_***lac*UV5 **_promoter DNA fragment cloned in pMW-P_***lac*UV5**_-lacI-118. Primer P2 is complementary to the 3'-terminus of the *lacI *gene and contains BamHI restriction site at the 5'-end. The amplified DNA fragment contained internal XbaI recognition site between the promoter and RBS of Φ10 gene of T7 phage providing translation of *lacI *gene in the pMW-P_***lac*UV5**_-lacI-118 plasmid. 5'-terminal portion of the *repB *gene of RSF1010 was amplified by PCR using primers P3 and P4. To provide translation of *repB *gene in the absence of the proximal *mobB *gene, the translation initiation region of the *repB *gene was modified by the addition of 4 nucleotides into the primer P3. Moreover, primers P3 and P4 contain *Bam*HI and *Kpn*I recognition sites at their 5'-ends, respectively. The two PCR products obtained were gel-purified, digested with *Bam*HI restriction endonuclease, ligated and used as a template for PCR with primers P1 and P4. The resulting DNA fragment was treated with *Xba*I and *Kpn*I restrictases and ligated with pBluescriptII-SK(+) vector treated with the same enzymes. The resulting plasmid was named pBluescript::lacIrepB.

In parallel, the fragment of the plasmid pACYC184 containing *cat *gene was amplified using primers P5 and P6. Primer P5 contains a *Bgl*II restriction site at the 5'-end necessary for removing of the *cat *marker gene from the plasmid after integration. Primer P6 contains a *Sac*I restriction site at the 5'-end. The P_***lac*UV5 **_promoter was amplified using primers P7 and P8 and the pMW-P_***lac*UV5**_-lacI-118 plasmid as a template. Primers P7 and P8 contain *Sac*I and *Xba*I restriction sites at their 5'-ends, respectively. The obtained fragments were gel-purified, treated with *Sac*I restrictase, ligated and used as a template for PCR with primers P5 and P8. The resultant PCR product containing the chloramphenicol resistance gene (*cat *gene) and the P_***lac*UV5 **_promoter was digested with *Xba*I restrictase and ligated with the pBluescript::lacIrepB plasmid pre-treated by the same restrictase. The obtained linear product was used as a template for PCR with primers P4 and P9. Primer P9 contains 38 nucleotides of the RSF1010 region, which is located between *oriV *and the 3'-end of the *mobC *gene, *Bgl*II restriction site and 17 nucleotides complementary to the 5'-end of the fragment comprising *cat *gene. As a result, the integrative DNA fragment containing P_***lac*UV5**_-*lacI *element linked to the sub-optimal SD sequence (GGGGGG), marked by *cat *gene and flanked by sequences homologous to the target sites of RSF1010 has been generated.

### λRed-mediated integration

Preparation of the electrocompetent cells was as in [23]. 100–200 ng of the PCR-amplified DNA fragment and 100 ng of the RSF1010 plasmid were used for electroporation. Electroporation was performed using Gene Pulser apparatus (BioRad, USA, version 2–89). Pulse time was 5 msec, electric field strength was 12.5 kV/cm. 1 ml of SOC medium was added to the cell suspension immediately after electroporation. The cells were cultivated at 37°C for 2 hours, spread on LB agar containing 30 μg/ml of chloramphenicol and cultivated at 37°C overnight.

### Determination of mobilization frequencies

Mobilization frequency of RSF1010 and RSFmob was calculated according to [34] as a number of transconjugants per recipient after overnight mating on LB-plates of the donor strain C600/RP1.2 harboring the RSF1010 or RSFmob plasmid and the recipient strain MG1655. Transconjugants were selected on M9 plates with streptomycin (50 mg/L).

### Estimation of the relative copy numbers

Estimation of the relative copy numbers of the constructed derivatives of RSF1010 plasmid has been performed mainly as in [15]. RSF1010, RSFmob and RSFmob-I plasmids were separately introduced to *E. coli *strain MG1655. The plasmid cells were cultivated in LB medium containing streptomycin (50 mg/L). If necessary, 1 mM IPTG was added. Plasmid DNA was isolated from the equal quantities of the cells grown overnight (stationary phase) or in 3 hours (logarithmic growth phase) using "GenElute Plasmid Miniprep Kit" (Sigma, USA), treated with EcoRV restrictase and RNAse A and resolved in 1% agarose gel. Copy numbers of the plasmids were estimated using "Sorbfil TLC Videodensitometer" software (ZAO "Sorbpolimer") by scanning of the electrophoretic bends corresponding to the large EcoRV fragments of each plasmid after coloration of the agarose gel with ethidium bromide. Three independent transformants of each type were analyzed.

### Electroporation of *Pantoea ananatis*

Overnight culture of *P. ananatis *SC17 strain grown in LB broth at +34°C was diluted 1:100 by fresh LB broth and cultivated at vigorous aeration up to OD_595 _= 0.8. Cells from 10 ml of the culture were collected by centrifugation at +4°C, washed three times by the same volume of ice-cold de-ionised water and after that by 1 ml of cold 10% glycerol. Cells were suspended in 40 μl of 10% glycerol and mixed with 100–200 ng of plasmid DNA dissolved in 1–5 μl of de-ionised water. Electroporation was performed using Gene Pulser apparatus (BioRad, USA, version 2–89). Pulse time was 5 msec; electric field strength was 20.0 kV/cm. 1 ml of LB broth was added immediately after electroporation. Cells were incubated at +34°C with aeration in 2 hours and plated on LB-agar with addition of 100 mg/L streptomycin.

## List of abbreviations

PCR: polymerase chain reaction. IPTG: isopropyl β-D-thiogalactopyranoside. bp: base pairs. Cm: chloramphenicol. Ap: ampicillin. Str: streptomycin. Rif: rifampicin.

## Authors' contributions

JIK designed the construction scheme and constructed RSFmobcat and RSFmob plasmids, checked plasmid stability and drafted the manuscript. TMK constructed RSFmob-I plasmid, performed plasmid copy number evaluation and helped to draft the manuscript. IGA introduced RSFmob to *P. ananatis *and checked its stability in this host. ILT performed the experiments with *M. methylotrophus*. AYS and IVB performed the experiment on plasmid mobilization. LIG helped in construction of RSFmob plasmid and figures preparation. SVM supervised and coordinated the work and edited the manuscript. All authors read and approved the final manuscript.
